# Yeast Diversity in Wine Grapes from Japanese Vineyards and Enological Traits of Indigenous *Saccharomyces cerevisiae* Strains

**DOI:** 10.3390/microorganisms12091769

**Published:** 2024-08-26

**Authors:** Kaito Shibayama, Kozue Kondo, Misa Otoguro

**Affiliations:** The Institute of Enology and Viticulture, University of Yamanashi, 1-13-1 Kitashin, Kofu 400-0005, Japan; shibayama-kaito@nite.go.jp (K.S.);

**Keywords:** yeast diversity, isolation, wine grape, non-*Saccharomyces* yeast, indigenous *Saccharomyces cerevisiae*

## Abstract

Japan has numerous vineyards with distinct geographical and climatic conditions. To the best of our knowledge, there is no comprehensive analysis of the diversity of yeasts associated with wine grapes from Japan. This study aimed to determine yeast diversity in wine grapes from four wine-producing regions in Japan and to evaluate the physicochemical characteristics of wines produced with indigenous *Saccharomyces cerevisiae* strains isolated from two regions. A total of 2648 strains were isolated from nine wine grape samples. MALDI-TOF MS and 26S rDNA sequence analyses revealed that the strains belonged to 21 non-*Saccharomyces* yeasts and 1 *Saccharomyces* yeast (*S. cerevisiae*). Non-*Saccharomyces* yeasts were found in high quantities and were highly distributed among the wine grape samples. Differences in the distribution of the identified yeast species were noted among the different wine grape varieties and regions. Indigenous *S. cerevisiae* strains of different genotypes from different regions exhibit distinct physiological traits. Our findings are expected to enhance our understanding of the local yeasts associated with Japanese vineyards and contribute to obtaining cultures that can provide region-specific organoleptic characteristics to local wines produced in Japan.

## 1. Introduction

One of the main sources of indigenous yeasts is the wine grape berry [1]. Numerous species/strains of indigenous yeasts are found on the berry surface and transferred to grape juice during winemaking. Indigenous yeasts are important for spontaneous fermentation and contribute to the complexity of wine production. *S. cerevisiae* and non-*Saccharomyces* strains such as *Candida*, *Hanseniaspora*, *Metschnikowia*, *Pichia*, *Torulaspora*, and *Zygosaccharomyces* have been isolated from wine grape berries and must from wine-producing regions [2,3,4,5,6,7,8,9,10,11,12,13]. The biodiversity and distribution patterns of wine grape yeasts vary by vineyard and region and are related to the geographical environment [1,14]. Moreover, indigenous wine grape yeasts may contribute to regional differences in wine and are part of terroirs, reflecting the typical characteristics of the region in which wine grapes are grown [15,16,17,18]. Terroir refers to the environment (soil, climate, topography, etc.) that gives a unique character to wine; it plays an important role in producing wines with region-specific organoleptic characteristics. Indigenous yeasts are also potential contributors to the unique characteristics and distinctive sensory attributes of wine.

The genetic diversity of *S. cerevisiae* associated with different vineyards contributes to the distinctiveness of wine phenotypes [19,20]. Several studies have reported the genetic diversity of *S. cerevisiae* and the preservation of bioresources for winemaking [21,22,23,24,25,26,27], both essential for maintaining the typical sensory properties of wines. This is because *S. cerevisiae* strains play a key role in providing distinctive organoleptic characteristics to wine produced via spontaneous fermentation.

Japan has numerous viticultural regions with distinct geographical and climatic conditions; thus, different yeast flora potentially exist in viticultural environments. Previous studies have reported the diversity of yeast strains isolated from wine grapes and must from several wine-producing areas of Japan, including Hiroshima, Okayama, Nagano, Yamanashi, Yamagata, and Iwate [28,29,30,31,32,33,34,35]. Yeast species in these wine-producing regions include the genera *Candida*, *Cryptococcus*, *Hanseniaspora*, *Kloeckera*, *Metschnikowia*, *Pichia*, *Rhodotorula*, and *Saccharomyces*. Indigenous yeasts from distinctive regions of Japan are considered parts of the terroir and can produce wines with region-specific organoleptic characteristics. However, to our knowledge, no comprehensive studies have been conducted on the yeast diversity in wine grapes from Japan. More information on the local yeast community in wine grapes is needed to evaluate the potential use of yeasts in winemaking and to produce high-quality wines with region-specific organoleptic characteristics.

Herein, we report the isolation, identification, and diversity of indigenous yeasts from wine grapes and spontaneous fermentation in vineyards located in four wine-producing regions in Japan. We also characterized the alcoholic fermentative profile of selected *S. cerevisiae* strains in laboratory fermentations. Our findings are expected to improve our understanding of yeast diversity and distribution in wine-producing regions in Japan and enable the preservation of potential wine yeasts for producing regionally differentiated wines, which may contribute to a better understanding of microbial terroirs in Japan.

## 2. Materials and Methods

### 2.1. Wine Grape Samples

**Figure 1:**
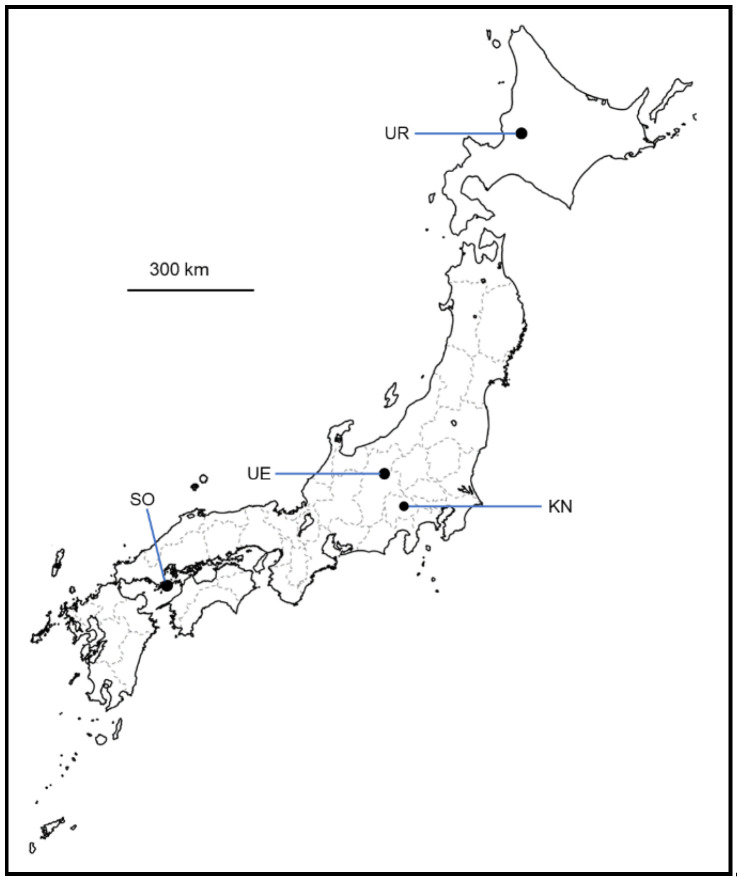
Geographic information of vineyards sampled in Japan. Maps were downloaded from https://www.freemap.jp/ (accessed on 30 November 2023) free of charge and modified using Microsoft PowerPoint software ® 2402. Abbreviations: UR, Urausu (Hokkaido); UE, Ueda (Nagano Prefecture); SO, Suo-Oshima (Yamaguchi Prefecture); KN, Katsunuma (Yamanashi Prefecture).

In this study, nine grape samples of the 2021 vintage were collected from vineyards located in four regions of Japan (Figure 1). The following grape varieties were used: Yama Sauvignon (YS), Yama Blanc (YB), Zweigelt (ZG), Pinot Blanc (PB), Chardonnay (CH), Merlot (MR), and Cabernet Sauvignon (CS) (Table 1). Healthy, undamaged grapes were collected from each sample, aseptically stored in sterile plastic bags, cooled using an ice pack, and transported to the Institute of Enology and Viticulture, University of Yamanashi, Yamanashi, Japan. The enological parameters of the resulting grape juice (pH, sugar content, and total acidity) were determined following the standard method in Japan [36] and are shown in Table 2. The sugar content was determined via conversion from specific gravity data.

### 2.2. Yeast Isolation

Yeast was isolated from the fermenting samples. Briefly, grape samples were manually destemmed and crushed in a sterile plastic bag. The must was fermented with the skin and seeds under microaerobic conditions in a sterilized 500 mL flask and incubated at 25 °C for 14–21 days. Samples (1 mL) were collected immediately after crushing the grapes and during spontaneous fermentation on days 2, 4, 7, 14, and 21. The fermenting samples were diluted with 0.85% NaCl (from 10^−1^ to 10^−5^). Then, 100 µL of these dilutions were spread onto YPD agar supplemented with 100 µg/mL chloramphenicol to inhibit bacterial growth. YPD agar plates were incubated at 25 °C until colonies formed. Colonies showing different morphologies were isolated from each YPD agar plate and then preserved at −80 °C in 10% glycerol.

### 2.3. Matrix-Assisted Laser Desorption/Ionization Mass Spectrometry (MALDI-TOF MS) Analysis

Rapid and tentative identification of yeast isolates was performed using a MALDI-TOF mass spectrometer (Shimadzu, Kyoto, Japan). The isolates were cultivated for two days on a YPD agar plate at 25 °C, and one loop of a colony (approximately 10 mg) was suspended in 300 µL distilled water and 900 µL ethanol. The suspension was centrifuged for 2 min at 16,000× *g*. The supernatant was discarded, and the pellet was air-dried. For protein extraction, 50 µL of 70% formic acid was added to the pellet and mixed, and then 50 µL of acetonitrile was added to the resulting mixture. The centrifuge tube containing the dissolved pellet in acetonitrile was centrifuged for 2 min at 13,000 rpm, and 1 µL of the protein solution was spotted on a target plate (Shimadzu) and left to dry. Each spot was overlaid with 1 µL of α-cyano-4-hydroxycinnamic acid matrix solution (Shimadzu) and then air-dried at 25 °C before analysis. Mass spectra of each isolate were automatically acquired using an AXIMA Performance mass spectrometer (Shimadzu). Tentative identification of the yeast isolates was performed using SARAMIS software version 4.04 (AnagnosTec, Potsdam, Germany). To temporarily identify yeast isolates, the obtained mass spectra were compared with those of known yeast species available in the in-house library database of the Institute of Enology and Viticulture at the University of Yamanashi. Next, yeast isolates were preliminarily grouped according to their mass spectral patterns via cluster analysis using SARAMIS software version 4.04, and 139 isolates were selected for molecular identification based on their 26S rDNA D1/D2 domain sequences.

### 2.4. 26S rDNA D1/D2 Domain Sequence Analysis

Identification was confirmed via sequence analysis. Genomic DNA of the 139 representative isolates was extracted using PrepMan™ Ultra Sample Preparation Reagent (Thermo Fisher Scientific, Waltham, MA, USA) according to the manufacturer’s protocol. The sequences of the D1/D2 domains of the 26S rDNA genes were amplified via PCR using primers NL1 (5′-GCATATCAATAAGCGGAGGAAAAG-3′) and NL4 (5′-GGAAGTAAAAGTCGTAACAAGG-3′) [37]. The PCR products were purified using a MonoFas DNA Purification Kit (GL Sciences, Tokyo, Japan) and sent to Fasmac Co., Ltd. (Kanagawa, Japan) for DNA sequence analysis with the same primers used for amplification. The obtained sequences were analyzed and compared with the sequences of the type strains using a BLASTN search (https://blast.ncbi.nlm.nih.gov/Blast.cgi (accessed on 11 December 2023)), considering an identity threshold of at least 98%.

### 2.5. Inter-Delta Analysis

In this study, 112 *S. cerevisiae* isolates (isolated from only two samples: MR in Katsunuma and CS in Ueda) were preliminarily classified based on their mass spectra patterns via cluster analysis using SARAMIS software version 4.04. A total of 23 representative isolates were selected for subsequent analysis. Genetic characterization of representative *S. cerevisiae* isolates was carried out via inter-delta analysis using a modified method with primers delta12 and delta21, based on a previous study [38]. The PCR products were separated via electrophoresis on 2.0% agarose gels, applied at 100 V for 130 min in 1× TBE buffer, and photographed under UV light. The band pattern obtained from each isolate was clustered using CLIQS 1D Pro analysis software version 1.5 (TotalLab Ltd., Newcastle upon Tyne, UK), and the unweighted pair group method using arithmetic averages was performed to construct a dendrogram. Finally, four *S. cerevisiae* isolates with different band patterns were selected and used for subsequent fermentation tests.

### 2.6. Laboratory-Scale Alcoholic Fermentation Using Selected Indigenous S. cerevisiae Isolates

To evaluate the enological traits of indigenous *S. cerevisiae* isolates, we carried out a fermentation test on four selected *S. cerevisiae* isolates in autoclaved grape juice derived from Muscat Bailey A (*Vitis labrusca* × *Vitis vinifera*), one the most popular Japanese wine grapes (pH 3.29; titratable acidity 6.56 g/L, as tartaric acid). The yeast assimilable nitrogen (YAN) of the grape juice was adjusted to 250 mg/L using diammonium phosphate and the sugar content was adjusted to 21% using sucrose. After adjusting the YAN and sugar content, the grape juice was autoclaved at 105 °C for 10 min. Yeast was cultured in sterile grape juice at 25 °C, and the culture was added to 100 mL of the grape juice (final cell density of approximately 10^6^ cells/mL). The inoculated grape juice was fermented for two weeks using a Fermograph II-W instrument (ATTO, Tokyo, Japan) at 25 °C. The fermentation process was evaluated by monitoring the CO_2_ produced every 120 min for two weeks. A commercial strain, *S. cerevisiae* EC1118 (Lallemand Inc., Montreal, QC, Canada), was used as a control. The physicochemical characteristics of the wine were quantified at the end of the alcoholic fermentation. Each strain was fermented in triplicate.

### 2.7. Physicochemical Analysis of Wine Samples

The ethanol content, pH, and total acidity of the wine samples derived from the fermentation of each *S. cerevisiae* isolate were determined following the standard method in Japan [36].

### 2.8. Gas Chromatography (GC) and High-Performance Liquid Chromatography (HPLC)

Using the internal standard method, the low-boiling-point aroma components of the wine samples were analyzed by directly injecting the wine samples into a gas chromatograph (GC; GC2014, Shimadzu) equipped with a flame ionization detector (FID). A PEG600-packed column (15% Chromosorb W 60/80 mesh, 2 m × 3 mm i.d.) was used for separation. Nitrogen was used as the carrier gas, and the flow rate was kept constant at 30 mL/min. The temperature of the GC oven was set at 95 °C. The injector and detector temperatures were set at 180 °C and 185 °C, respectively. Briefly, 2 mL of the wine sample and 0.2 mL of an internal standard (acetaldehyde, ethyl acetate, n-propyl alcohol, isobutyl alcohol, isoamyl alcohol, or ethyl lactate) were added to a 5 mL test tube. The test tube was vortexed, and 1 µL of each sample was injected into the GC.

The intermediate- and high-boiling-point aroma components were quantified using the internal standard method by directly injecting wine samples into a GC (GC2014, Shimadzu) equipped with an FID and an autosampler. The aroma components were separated on a Pure-WAX column (GL Sciences Inc., Tokyo, Japan; 0.25 mm i.d. × 60 m, 0.25 µm film thickness) using helium carrier gas at a constant 1 mL/min flow rate. The temperature of the GC oven was initially set to 50 °C for 5 min, raised to 130 °C at a rate of 4 °C/min, further increased to 220 °C at a rate of 5 °C/min, and maintained at 220 °C for 12 min. The injector and detector temperatures were set to 250 °C and 260 °C, respectively. Briefly, 40 mL of the wine sample, 2 g ammonium sulfate, 0.4 mL of an internal standard (2-octanol, ethyl propanoate, ethyl butyrate, isoamyl acetate, ethyl hexanoate, n-hexyl acetate, ethyl lactate, n-hexyl alcohol, (Z)-3-hexen-1-ol(cis), ethyl octanoate, propionic acid, i-butyric acid, butanoic acid, ethyl decanoate, i-valeric acid, diethyl succinate, methionol, 2-phenethyl acetate, hexanoic acid, 2-phenethyl alcohol, octanoic acid, or decanoic acid), and 10 mL of pentane/diethyl ether (1:1) were added to a 50 mL glass centrifuge tube. The centrifuge tube was inverted for 5 min and then maintained at 15 °C for 5 min. After inversion twice, the sample was centrifuged at 1700× *g* for 20 min. The supernatant was transferred into a 2 mL vial, and 1.5 µL of the solution was injected into the GC. The split ratio was 1:15.

Organic acids in the wine samples were quantified using a Shimadzu LabSolutions HPLC system consisting of a Shim-pack SCR-102H column and an autosampler (AS-2000). The wine samples were diluted 1:10 with water (Milli-Q) and passed through a membrane filter (0.22 μm). The filtrate was transferred into a 1.5 mL vial tube. Samples were injected into the column at a 0.8 mL/min flow rate. Quantification was performed using calibration curves (R2 > 0.99) prepared with organic acid standards.

### 2.9. Statistical Analysis

The chemical compounds in the wine samples obtained via fermentation with indigenous *S. cerevisiae* strains were subjected to a one-way analysis of variance to evaluate the differences in physicochemical characteristics followed by the Tukey–Kramer test at a significance level of *p* < 0.01. The results are expressed as the means ± standard deviations.

## 3. Results

### 3.1. Isolation and Identification of Yeast Species

A total of 2648 yeast isolates were collected from nine grape samples collected from four different regions of Japan. The number of isolates and the physicochemical characteristics of the grape samples are listed in Table 2. The pH levels ranged from 2.91 to 3.84, the sugar contents ranged from 15.9 to 20.2%, and the total acidity ranged from 3.96 to 8.28 g/L.

**Figure 2:**
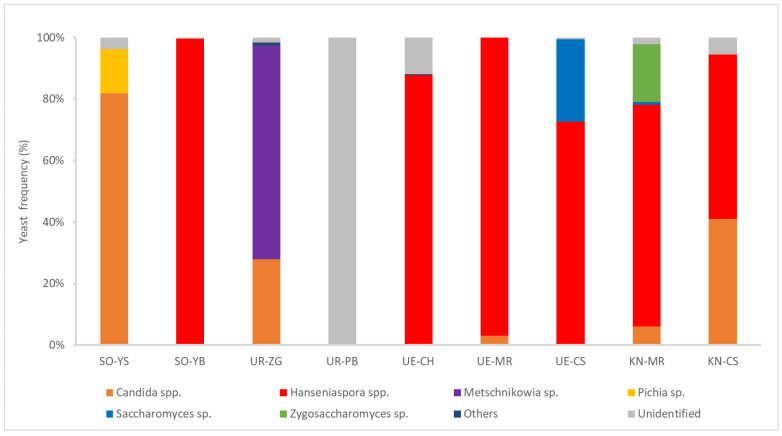
Distribution frequencies of yeasts isolated from wine grapes in four wine-producing regions, as determined using MALDI-TOF MS.

All yeast isolates were analyzed via MALDI-TOF MS for rapid and tentative identification. In this study, 157 yeast isolates from PB in Urausu could not be identified because their MS data did not correspond to the reference data. The yeast genera observed included *Candida*, *Hanseniaspora*, *Metschnikowia*, *Pichia*, *Saccharomyces*, and *Zygosaccharomyces* (Figure 2). *Hanseniaspora* was the most abundant and frequently isolated genus, identified in six samples. *Saccharomyces* was detected in only two samples (CS from Ueda and MR from Katsunuma). *Metschnikowia*, *Pichia*, and *Zygosaccharomyces* were also detected in only one sample. Although these results were tentative, they revealed a high overall diversity of non-*Saccharomyces* yeasts in wine grapes collected from different regions of Japan.

Table S1 shows the identification results for the 139 representative yeast isolates based on 26S rDNA D1/D2 domain sequence analysis. Most yeast isolates were identified to have a high similarity (>99%) with the type strains of known species. A total of 22 species (16 genera) were detected: *Aureobasidium pullulans*, *Clavispora* sp., *Hanseniaspora guilliermondii*, *Hanseniaspora opuntiae*, *Hanseniaspora uvarum*, *Hanseniaspora valbyensis*, *Hanseniaspora vineae*, *Lachancea thermotolerans*, *Martiniozyma asiatica*, *Metschnikowia shanxiensis*, *Meyerozyma caribbica*, *Papiliotrema laurentii*, *Pichia manshurica*, *Rhodotorula graminis*, *Rhodotorula nothofagi*, *Saccharomyces cerevisiae*, *Saturnispora diversa*, *Sporidiobolus pararoseus*, *Starmerella apicola*, *Starmerella bacillaris* (syn. *Candida zemplinina*), *Torulaspora delbrueckii*, and *Zygosaccharomyces bailii* (Table 3). The PB isolates from Urausu, which could not be identified via MALDI-TOF MS, were identified as *R. nothofagi* and *S. pararoseus* based on 26S rDNA sequence analysis.

Differences in the distribution of the identified yeast species were observed among different grape varieties collected from the same region. In Suo-Oshima, *M. caribbica* was the dominant species detected in YS samples, while *H. guilliermondii* was the most frequently isolated species in YB samples. In Urausu, the isolates from ZG and PB were also diverse. The most frequently isolated species from ZG was *M. shanxiensis* (69.4%), followed by *T. delbrueckii* (30.6%) and *R. nothofagi* (99.4%). In Ueda, the species most frequently isolated from CH, MR, and CS samples was *H. uvarum* (87.46%, 63.7%, and 62.47%, respectively). The second most frequently isolated species was *R. nothofagi* for CH (9.90%), *H. valbyensis* for MR (25.8%), and *S. cerevisiae* for CS (26.91%). The wine grapes from Katsunuma (MR and CS) also had a high abundance of *H. uvarum* (56.89% and 46.87%, respectively), followed by *Z. bailii* in MR (20.05%) and *S. bacillaris* in CS (41.60%). Sixteen species (*A. pullulans*, *Clavispora* sp., *H. opuntiae*, *H. valbyensis*, *L. thermotolerans*, *M. asiatica*, *M. shanxiensis*, *M. caribbica*, *P. laurentii*, *P. manshurica*, *R. graminis*, *S. diversa*, *S. pararoseus*, *S. apicola*, *T. delbrueckii*, and *Z. bailii*) detected in this study were exclusively found in only one sample. Our data also showed that the diversity of isolated indigenous yeasts in Japan differed between wine-producing regions. Although *H. uvarum* was the most abundant species in MR samples from two different regions (Ueda and Katsunuma), *H. valbyensis* was only the second most abundant species in MR samples from Ueda (25.8%); it was not found in the MR samples from Katsunuma. In addition, *H. uvarum* was the most abundant species in the CS from Ueda and Katsunuma, whereas *S. bacillaris* was isolated at a frequency of 41.60% from Katsunuma and only 0.25% from Ueda.

### 3.2. Inter-Delta Analysis of Indigenous S. cerevisiae Isolates

**Figure 3:**
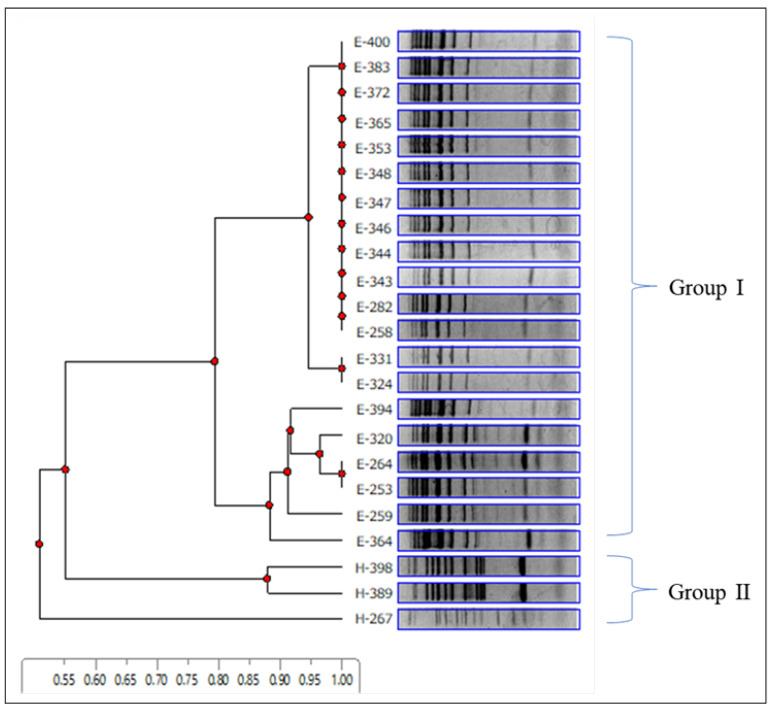
Inter-delta analysis of representative *Saccharomyces cerevisiae* strains isolated from Ueda and Katsunuma vineyards. Electrophoretic patterns and cluster analyses were performed using delta12 and delta21 primers, as described by Legras and Karst (2003) [38]. The dendrogram was constructed using an unweighted pair group method with an arithmetic mean. Group I comprised 20 isolates originating from Ueda with seven genotypes, and group II was composed of isolates from Katsunuma with three genotypes.

In this study, 112 isolates identified as *S. cerevisiae* were collected from wine grapes from Ueda and Katsunuma. Specifically, 23 representative isolates (20 isolates from a vineyard in Ueda and 3 isolates from Katsunuma) were selected for inter-delta analysis to determine their genetic diversity. Ten distinct genotypes were identified in this study. The band pattern data from the inter-delta analysis were used to construct a dendrogram (Figure 3). The cluster analysis showed that the 23 isolates diverged into two major groups at a 0.55 distance, suggesting that the two wine-producing regions have *S. cerevisiae* strains of different genotypes. Group I comprised 20 isolates originating from Ueda with seven genotypes, and group II was composed of isolates from Katsunuma with three genotypes.

### 3.3. Physicochemical Properties of Wines Produced Using Indigenous Yeasts

Four representative indigenous *S. cerevisiae* isolates with different genotypes, E-253 and E-400 isolated from a vineyard in Ueda and H-267 and H-389 from Katsunuma, were used for fermentation tests with sterilized Muscat Bailey A juice to evaluate the enological traits of these isolates. *S. cerevisiae* EC1118 (Lallemand, Inc.) was used as a control. Table 4 shows the physicochemical characteristics of the wines produced via fermentation with these isolates. Our results showed that the physicochemical characteristics varied depending on the strain. A comparison of wines produced using indigenous *S. cerevisiae* isolates with different genotypes from Ueda (E-253 and E-400) and Katsunuma (H-267 and H-389) revealed that the concentrations of 16 of the 28 wine components investigated were significantly different (*p* < 0.01). Among these components, the concentrations of lactic acid, acetic acid, isobutyl alcohol, ethyl octanoate, and decanoic acid significantly differed between the wines produced using the two Katsunuma isolates and those produced using the two Ueda isolates.

The alcohol content and pH values of the wines obtained after fermentation for two weeks were similar. In contrast, the total acidity and lactic and acetic acid concentrations significantly differed between wines produced with isolates from Ueda (E-253 and E-400) and those from Katsunuma (H-267 and H-389). The total acidity and lactic acid concentrations in wines produced via fermentation with the Katsunuma isolates (H-267 and H-389) were significantly higher than those produced with EC1118. Similarly, the acetic acid concentration in wines fermented with the two Ueda isolates (E-253 and E-400) was significantly higher than in wines fermented with EC1118.

Nineteen aromatic components were identified and quantified using GC. Significant differences also occurred in the low-boiling-point aroma components of the wines produced. Wines produced with the Ueda isolates (E-253 and E-400) had higher acetaldehyde concentrations than those derived from Katsunuma isolates (H-267 and H-389), and significant differences in acetaldehyde concentrations were noted between wines produced with E-253 and H-389 and those produced with E-400 and H-389. All wines produced using the indigenous isolates had lower acetaldehyde and ethyl acetate concentrations than those produced using EC1118. The concentrations of isobutyl and isoamyl alcohols in the wines produced with H-267 were significantly higher than those in the other wines. All low-boiling-point aroma components in the wines produced using E-253 and E-400 had lower concentrations than those produced using EC1118.

Regarding the intermediate- and high-boiling-point aroma components, the ethyl octanoate and decanoic acid concentrations significantly differed between wines obtained via fermentation with the two Katsunuma isolates and those obtained via fermentation with the two Ueda isolates. Most intermediate- and high-boiling-point aroma components in wines produced with indigenous isolates were more highly concentrated than those in the control wine. The concentration of isoamyl acetate, known for its fruity aroma, was higher in wines fermented with H-267 and H-389 than in those fermented with EC1118. The wine produced by fermentation with H-389 had a higher concentration of ethyl hexanoate than that produced by fermentation with EC1118. Wines produced using all four indigenous isolates (E-253, E-400, H-267, and H-389) had higher 2-phenethyl alcohol concentrations than those produced using EC1118.

## 4. Discussion

Yeast species from the genera *Hanseniaspora*, *Metschnikowia*, and *Saccharomyces*, particularly *H. uvarum*, *M. pulcherrima*, and *S. cerevisiae*, respectively, are commonly found in wine grapes and wines in Japan [28,30,32,33,34] but have not been well-studied in the past 20 years. In this study, 2648 isolates were collected from nine grape samples collected from four wine-producing regions in Japan. All isolates were initially analyzed via MALDI-TOF MS for rapid identification, revealing that the isolates belonged to six major yeast genera. Non-*Saccharomyces* species were detected at high frequencies in all grape samples, whereas *Saccharomyces* species were detected in only two samples. 

The identification of 22 yeast species belonging to 16 genera isolated from nine grape samples via 26S rDNA sequence analysis (Table 3) indicated that non-*Saccharomyces* yeast species were dominant, consistent with previous studies in Japan [28,32] and other countries [2,5,8,39,40]. Although non-*Saccharomyces* yeasts are less capable of fermenting sugars in juice than *Saccharomyces* yeasts [41,42], they can produce secondary metabolites that contribute to the diversity and complexity of wine, and research has focused on their application to winemaking [41,43]. Recently, commercial starter cultures containing non-*Saccharomyces* species and strains have been used in winemaking [44].

*H. uvarum* is the most abundant species isolated from wine grapes in Ueda and Katsunuma. *H. uvarum* is known to be frequently isolated from wine grapes [1,4,8,32,45]. The effect of *H. uvarum* on the physicochemical characteristics of wine has also been studied [45,46]. Exploring the potential of starter cultures containing indigenous *H. uvarum* strains in the local winemaking industry is important. *H. vineae* is also commonly isolated from wine grape samples in Ueda and Katsunuma. *H. vineae* improves wine aroma [47]. A previous sensory analysis demonstrated a significant increase in the fruity aroma evoking bananas, pears, apples, citrus fruits, and guava in wine produced using *H. vineae* [48]. The *H. vineae* strains isolated in this study should be further analyzed for their potential to produce wines with a characteristic aroma and flavor. Other isolated *Hanseniaspora* species include *H. guilliermondii*, *H. opuntiae*, and *H. valbyensis*. This is the first study to identify *H. opuntiae* and *H. valbyensis* in wine grapes in Japan. 

In contrast, *S. bacillaris* (synonym: *C. zemplinina*) was the predominant species in CS samples from Katsunuma. *C. zemplinina* has been previously isolated from wine grapes in Japan [34], and its application in winemaking has been investigated [49,50]. We are the first to isolate and identify *Z. bailii* from wine grape samples from Katsunuma. Although this species is a spoilage yeast responsible for off-flavors and sediment formation [51], it produces high levels of esters and shows potential as a co-starter during fermentation with *S. cerevisiae* [52]. *M. caribbica* is the most abundant yeast species in YS samples from Suo-Oshima and is a new species found in wine grapes in Japan. *M. caribbica* is commonly found in wine grapes in Italy, Spain, and other regions [5,8,53]. *M. shanxiensis* was isolated from only one wine grape sample (UR-ZG). This species was first isolated from the surfaces of jujube fruits collected in China [54]. This is the first report of *M. shanxiensis* isolated from wine grapes and wine-producing environments. 

*T. delbrueckii* was isolated only from wine grapes in Urausu. One of the most well-studied non-*Saccharomyces* yeasts, *T. delbrueckii* is commercially available for wine production [44]. *R. nothofagi* was the dominant species isolated from PB samples from Urausu, a region with a cold climate. The basidiomycetous yeast genus *Rhodotorula* is found in wine grapes in Japan (*Rhodotorula glutinis* and *R. minuta*) [32]. *R. nothofagi* was the third *Rhodotorula* species isolated from wine grapes in Japan. In addition, *A. pullulans*, *Clavispora* sp., *L. thermotolerans*, *P. laurentii*, *R. graminis*, *S. diversa*, *S. pararoseus*, and *S. apicola* were isolated at very low frequencies (0.49%, 2.5%, 0.33%, 0.6%, 1.65%, 0.25%, 0.6%, and 0.25%, respectively) from one sample only, which led us to speculate that their presence on wine grapes was probably incidental. These results show the data of a single year’s isolation. To have more comprehensive data on the yeast diversity at the species level in wine grapes in Japan, further studies on the isolation and identification of yeasts carried out in different years and different regions will be necessary.

Our study offers new insights into yeast diversity in the wine-producing regions of Japan. Future work should evaluate the contribution of indigenous non-*Saccharomyces* isolates from Japanese wine grapes to wine aroma and discover new strains with excellent enological traits. The wine-grape-associated yeast community is altered by climatic conditions, soil, and cultivars [1,14]. Our finding that indigenous yeast species differ by region indicates that yeast diversity in wine grapes from Japan may depend on the grape variety or vineyard location. Different yeast distributions in different regions may contribute to producing wine with distinctive flavors [15,55].

Non-*Saccharomyces* yeasts were found in high quantities and were widely distributed in the wine grape samples examined in this study. In contrast, *S. cerevisiae* isolates were found in only two grape samples (CS in Ueda and MR in Katsunuma). *S. cerevisiae* has an extremely low abundance in wine grapes [8,56,57]. In contrast, *S. cerevisiae* was the predominant species when self-enrichment (spontaneous fermentation) was performed [53]. Although self-enrichment was performed in this study, *S. cerevisiae* was still detected in low abundance. Different approaches should be considered to increase the abundance of isolated *S. cerevisiae* in the future, such as adding SO_2_, fermentation temperature control, and maintaining anaerobic conditions to suppress non-*Saccharomyces* yeast from becoming dominant [35].

In a previous study, *S. cerevisiae* strains were isolated from vineyard soils, grapes, grape juices, and grape pomace in the Yamanashi area and classified into 35 karyotypes [33]. In this study, indigenous *S. cerevisiae* isolates were chosen for further genotypic characterization and the evaluation of their enological traits. The dendrogram from the inter-delta analysis suggested that the two wine-producing regions contained *S. cerevisiae* strains of different genotypes. The *S. cerevisiae* isolates in this study resulted from a single year of isolation, and further isolation in consecutive vintages is needed for reliable genotypic characterization, similar to other studies conducted using consecutive vintages [10,26]. In addition, these strains were not isolated from the same grape varieties; therefore, the influence of the grape variety should also be considered. Further studies are required to determine whether these indigenous *S. cerevisiae* isolates contribute to the typicity of wines from these regions. 

The isolated *S. cerevisiae* strains exhibited interesting physiological characteristics. Our results show, for the first time, that indigenous yeasts with different genotypes originating from different wine-producing regions in Japan have distinctive enological traits. In this study, we prefer to focus on investigating the differences in the enological traits of indigenous *S. cerevisiae* strains rather than enological points for our strains being suitable for application in winemaking. Therefore, our results suggest preliminary data and initial steps for application in winemaking. It will be needed to measure reducing sugars and glycerol, which are important parameters for wines, to add SO_2_, and to investigate fermentation dynamics. Further analyses are necessary to verify the use of these strains in winemaking with final sensorial evaluation. Moreover, further studies to investigate the organoleptic characteristics that native yeasts bring to wines produced in different regions using must from various grape varieties and the grapes from which the yeasts were isolated are required. Non-*Saccharomyces* strains were not included in the fermentation tests in this study. Genotypic and phenotypic characterizations of non-*Saccharomyces* strains are needed to improve our understanding of yeast diversity in Japan and the preservation of potential wine yeasts, considering their high quantity and high distribution among the samples. Further investigations on the enological traits of non-*Saccharomyces* strains will be necessary.

## 5. Conclusions

We studied the diversity and distribution of yeast associated with wine grapes from vineyards in Japan. There is a lack of information on such yeast, so our findings provide new information on the yeast flora in the grape-growing regions of Japan. Wine grapes in Japan are the principal source of different yeast species, particularly non-*Saccharomyces* species. However, the evaluation of indigenous *S. cerevisiae* isolates revealed that these strains have different enological traits, and region-specific yeast strains may exist and contribute to the wine terroir. Our findings will contribute to a better understanding of local yeasts associated with these terroirs and the use of starter cultures to provide region-specific organoleptic characteristics to local wines.

## Figures and Tables

**Table 1:** Geographic information of sampling points from vineyards in Japan.

Sample ID	Grape Variety	Date Collected	Location of Sampling Vineyard			
			Location	Latitude, Longitude	Altitude (m)	Precipitation in Year (mm)
SO-YS	Yama Sauvignon	5 September 2021	Suo-Oshima (Yamaguchi Prefecture)	33.903466, 132.313487	8	2112.5
SO-YB	Yama Blanc	5 September 2021	Suo-Oshima (Yamaguchi Prefecture)	33.905328, 132.314701	6	2112.5
UE-CH	Chardonnay	20 September 2021	Ueda (Nagano Prefecture)	36.344393, 138.302831	635	992.0
UR-ZG	Zweigelt	28 September 2021	Urausu (Hokkaido)	43.458333, 141.806111	110	1458.5
UR-PB	Pinot Blanc	28 September 2021	Urausu (Hokkaido)	43.463333, 141.804166	100	1458.5
UE-MR	Merlot	30 September 2021	Ueda (Nagano Prefecture)	36.344768, 138.303732	635	992.0
KN-MR	Merlot	4 October 2021	Katsunuma (Yamanashi Prefecture)	35.649633, 138.745118	580	1065.5
UE-CS	Cabernet Sauvignon	25 October 2021	Ueda (Nagano Prefecture)	36.346435, 138.305098	635	992.0
KN-CS	Cabernet Sauvignon	2 November 2021	Katsunuma (Yamanashi Prefecture)	35.649633, 138.745118	580	1065.5

**Table 2:** Physicochemical characteristics and number of yeast isolates obtained from the wine grape samples in this study.

Sample ID	Grape Variety	pH ^a^	Sugar Content (%)	Total Acidity (g/L) ^a^	No. of Isolates
SO-YS	Yama Sauvignon	3.65	16.7 ^c^	4.97	160
SO-YB	Yama Blanc	3.84	15.9 ^c^	5.85	212
UE-CH	Chardonnay	3.19	18.3 ^b^	6.16	303
UR-ZG	Zweigelt	3.04	19.4 ^c^	5.92	186
UR-PB	Pinot Blanc	2.91	16.7 ^b^	7.89	157
UE-MR	Merlot	3.33	19.1 ^b^	3.96	427
KN-MR	Merlot	3.43	19.4 ^b^	5.54	399
UE-CS	Cabernet Sauvignon	3.19	18.8 ^b^	5.42	405
KN-CS	Cabernet Sauvignon	3.48	20.2 ^b^	8.28	399

^a^ Data represent the average of three replicates. ^b^ Data represent the average of two replicates. ^c^ Data for only one measurement is shown.

**Table 3:** Yeast species and their percentage of frequency (%) isolated from wine grapes from four wine-producing regions in Japan.

Yeast Species	SO-YS	SO-YB	UR-ZG	UR-PB	UE-CH	UE-MR	UE-CS	KN-MR	KN-CS	Representative Isolates
*Aureobacidium pullulans*	-	-	-	-	-	-	2 (0.49)	-	-	E-4
*Clavispora* sp.	4 (2.5)	-	-	-	-	-	-	-	-	A-35, A-76
*Hanseniaspora guilliermondii*	-	160 (75.5)	-	-	-	5 (1.2)	-	-	-	B-48, B-54, B-106, B-166, B-168, B-180, D-111, D-192, D-298
*Hanseniaspora opuntiae*	-	13 (6.1)	-	-	-	-	-	-	-	B-137
*Hanseniaspora uvarum*	-	39 (18.4)	-	-	265 (87.46)	272 (63.7)	253 (62.47)	227 (56.89)	187 (46.87)	B-23, B-47, B-64, B-138, B-170, C-24, C-93, C-101, C-170, C-296, D-25, D-71, D-102, D-137, D-233, D-276, D-387, E-41, E-54, E-59, E-127, E-241, E-245, E-304, H-1, H-31, H-73, H-111, H-115, H-142, H-149, H-151, H-166, H-191, H-210, H-211, H-213, H-238, I-14, I-15, I-55, I-147, I-221, I-249
*Hanseniaspora valbyensis*	-	-	-	-	-	110 (25.8)	-	-	-	D-17, D-60, D-178, D-224, D-323, D-356
*Hanseniaspora vineae*	-	-	-	-	2 (0.66)	27 (6.3)	40 (9.88)	64 (16.04)	28 (7.02)	C-247, C-268, D-381, D-402, E-294, E-310, H-87, H-208, H-262, I-133, I-197, I-241, I-321
*Lachancea thermotorelans*	-	-	-	-	1 (0.33)	-	-	-	-	C-26
*Martiniozyma asiatica*	-	-	-	-	-	-	-	-	17 (4.26)	I-18, I-23, I-57, I-59, I-63
*Metschnikowia shanxiensis*	-	-	129 (69.4)	-	-	-	-	-	-	F-11, F-84, F-176
*Meyerozyma caribbica*	131 (81.9)	-	-	-	-	-	-	-	-	A-2, A-52, A-117
*Papiliotrema laurentii*	1 (0.6)	-	-	-	-	-	-	-	-	A-4
*Pichia manshurica*	24 (15.0)	-	-	-	-	-	-	-	-	A-72, A-136
*Rhodotorula graminis*	-	-	-	-	5 (1.65)	-	-	-	-	C-129, C-137, C-144
*Rhodotorula nothofagi*	-	-	-	156 (99.4)	30 (9.90)	-	-	-	-	C-29, C-32, C-123, C-138, G-10, G-29, G-45, G-144
*Saccharomyces cerevisiae*	-	-	-	-	-	-	109 (26.91)	3 (0.75)	-	E-324, E-365, H-267, H-389
*Saturnispora diversa*	-	-	-	-	-	-	-	1 (0.25)	-	H-221
*Sporidiobolus pararoseus*	-	-	-	1 (0.6)	-	-	-	-	-	G-72
*Starmerella apicola*	-	-	-	-	-	-	-	-	1 (0.25)	I-6
*Starmerella bacillaris* (syn. *Candida zemplinina*)	-	-	-	-	-	13 (3.0)	1 (0.25)	24 (6.02)	166 (41.60)	D-324, D-397, E-3, H-79, H-106, H-200, H-272, I-66, I-68, I-128, I-130, I-195, I-207, I-266, I-292, I-353, I-370, I-378
*Torulaspora delbrueckii*	-	-	57 (30.6)	-	-	-	-	-	-	F-1, F-26, F-34, F-47, F-48, F-146
*Zygosaccharomyces bailii*	-	-	-	-	-	-	-	80 (20.05)	-	H-281, H-359, H-366, H-367, H-368, H-388
Total	160	212	186	157	303	427	405	399	399	

-: Not detected. Abbreviations: SO, Suo-Oshima; UR, Urausu; UE, Ueda; KN, Katsunuma. YS: Yama Sauvignon; YB: Yama Blanc; ZG: Zweigelt; PB: Pinot Blanc; CH: Chardonnay; MR: Merlot; CS: Cabernet Sauvignon.

**Table 4:** Physicochemical characteristics of and concentrations of aroma components in wines produced by fermentation with indigenous *Saccharomyces cerevisiae* E-253 and E-400 isolated from Ueda (Nagano) and H-267 and H-389 isolated from Katsunuma (Yamanashi). *S. cerevisiae* EC1118 was used as a control.

Physicochemical Characteristic/Component	Yeast Strain				
	E-253 *	E-400 *	EC1118 ***	H-267 *	H-389 *
Alcohol (%)	11.82 ± 0.14 ^a^	11.96 ± 0.08 ^a^	12.05 ± 1.02 ^a^	12.59 ± 0.88 ^a^	12.75 ± 0.12 ^a^
pH	3.56 ± 0.02 ^a^	3.58 ± 0.03 ^a^	3.56 ± 0.16 ^a^	3.47 ± 0.02 ^a^	3.42 ± 0.02 ^a^
Total acidity (as tartaric acid g/L)	6.83 ± 0.08 ^b^	6.70 ± 0.08 ^b^	7.05 ± 0.29 ^b^	7.67 ± 0.09 ^a^	7.70 ± 0.17 ^a^
Organic acid (g/L)					
Citric acid	0.55 ± 0.02 ^a^	0.56 ± 0.01 ^a^	0.52 ± 0.05 ^a^	0.48 ± 0.01 ^a^	0.48 ± 0.01 ^a^
Tartaric acid	2.34 ± 0.22 ^ab^	2.25 ± 0.18 ^b^	2.70 ± 0.64 ^ab^	3.43 ± 0.09 ^ab^	3.63 ± 0.05 ^a^
Malic acid	2.27 ± 0.08 ^a^	2.32 ± 0.02 ^a^	2.51 ± 0.28 ^a^	2.24 ± 0.02 ^a^	2.13 ± 0.01 ^a^
Succinic acid	0.48 ± 0.03 ^a^	0.47 ± 0.01 ^a^	0.38 ± 0.07 ^ab^	0.19 ± 0.16 ^b^	0.48 ± 0.03 ^a^
Lactic acid	0.23 ± 0.02 ^b^	0.23 ± 0.01 ^b^	0.23 ± 0.01 ^b^	0.33 ± 0.01 ^a^	0.33 ± 0.00 ^a^
Acetic acid	0.54 ± 0.03 ^a^	0.55 ± 0.01 ^a^	0.20 ± 0.04 ^b^	0.24 ± 0.01 ^b^	0.23 ± 0.01 ^b^
Low-boiling-point aroma component (mg/L)					
Acetaldehyde	52.17 ± 4.15 ^ac^	56.29 ± 5.42 ^ac^	63.26 ± 10.53 ^a^	36.71 ± 1.83 ^bc^	23.49 ± 7.06 ^b^
Ethyl acetate	19.92 ± 4.81 ^b^	21.91 ± 1.90 ^b^	41.38 ± 4.64 ^a^	39.68 ± 4.57 ^ac^	27.32 ± 2.67 ^bc^
n-Propyl alcohol	46.76 ± 40.62 ^ab^	21.82 ± 0.68 ^b^	86.15 ± 2.41 ^a^	69.91 ± 7.73 ^ab^	45.71 ± 7.52 ^ab^
Isobutyl alcohol	17.75 ± 1.15 ^cd^	17.32 ± 0.10 ^c^	29.56 ± 7.97 ^bc^	135.04 ± 6.51 ^a^	46.09 ± 5.44 ^b^
Isoamyl alcohol	84.22 ± 8.07 ^bc^	80.93 ± 1.54 ^c^	101.75 ± 7.11 ^b^	134.91 ± 5.54 ^a^	92.96 ± 10.54 ^bc^
Intermediate- and high-boiling-point aroma component (mg/L)					
Ethyl butyrate	1.84 ± 0.07 ^ab^	1.75 ± 0.07 ^b^	1.98 ± 0.24 ^ab^	2.28 ± 0.04 ^a^	2.32 ± 0.03 ^a^
Isoamyl acetate	7.35 ± 0.39 ^a^	7.40 ± 0.14 ^a^	8.16 ± 1.08 ^a^	9.35 ± 0.10 ^a^	9.45 ± 0.14 ^a^
Ethyl hexanoate	0.36 ± 0.04 ** ^a^	0.23 ± 0.02 ^a^	0.39 ± 0.12 ^a^	0.33 ± 0.09 ^a^	0.67 ± 0.48 ^a^
Ethyl lactate	5.40 ± 0.71 ^a^	4.56 ± 1.13 ^a^	7.96 ± 1.75 **** ^a^	8.82 ± 1.81 ^a^	8.23 ± 3.63 ^a^
n-Hexyl alcohol	0.28 ± 0.09 ^a^	0.25 ± 0.04 ^a^	0.19 ± 0.03 ^a^	0.29 ± 0.03 ^a^	0.25 ± 0.02 ^a^
Ethyl octanoate	5.06 ± 0.13 ^a^	4.91 ± 0.21 ^a^	1.77 ± 0.42 ^b^	2.17 ± 0.04 ^b^	2.11 ± 0.14 ^b^
i-Butyric acid	1.42 ± 0.34 ^bc^	1.29 ± 0.12 ^bc^	1.15 ± 0.42 ^b^	4.16 ± 0.37 ^a^	2.20 ± 0.08 ^c^
Butanoic acid	0.55 ± 0.19 ^a^	0.72 ± 0.13 ^a^	0.79 ± 0.12 ^a^	0.98 ± 0.46 ^a^	0.85 ± 0.16 ^a^
i-Valeric acid	0.28 ± 0.04 ** ^a^	0.35 ± 0.06 ^a^	0.33 ± 0.07 **** ^a^	0.54 ± 0.23 ^a^	0.38 ± 0.02 ^a^
2-Phenethyl acetate	0.52 ± 0.03 ^a^	0.48 ± 0.02 ^a^	0.42 ± 0.09 ^a^	0.47 ± 0.07 ^a^	0.36 ± 0.05 ^a^
Hexanoic acid	2.47 ± 0.14 ^ab^	2.42 ± 0.11 ^ab^	2.53 ± 0.18 ^ab^	2.21 ± 0.07 ^b^	2.83 ± 0.10 ^a^
2-Phenethyl alcohol	22.09 ± 0.90 ^a^	21.13 ± 0.43 ^ac^	13.45 ± 3.25 ^b^	15.44 ± 0.36 ^ab^	14.40 ± 0.50 ^bc^
Octanoic acid	2.76 ± 0.14 ^b^	2.36 ± 0.14 ^c^	3.60 ± 0.10 ^a^	2.36 ± 0.15 ^c^	2.74 ± 0.07 ^bc^
Decanoic acid	2.20 ± 0.20 ^a^	1.87 ± 0.19 ^ab^	1.45 ± 0.31 ^bd^	0.62 ± 0.02 ^c^	0.91 ± 0.06 ^cd^

^a,b,c,d^ Different letters within the same row indicate significant differences between values using the Tukey–Kramer test (*p* < 0.01). * data represent means of three replicates ± SD; ** data represent means of two replicates ± SD; *** data represent means of two independent sets and three replicates (*n* = 6) ± SD; **** data represent means of five replicates ± SD.

## Data Availability

Data are contained within the article or Supplementary Material.

## Supplementary Materials

The following supporting information can be downloaded at: https://www.mdpi.com/article/10.3390/microorganisms12091769/s1, Table S1: Representative isolates and identified yeast species based on the sequencing of the 26S rDNA D1/D2 domain.
