# Supplementary material for: Yeast Diversity in Wine Grapes from Japanese Vineyards and Enological Traits of Indigenous Saccharomyces cerevisiae Strains
Source: Microorganisms. 2024 Aug 26;12(9):1769. doi: 10.3390/microorganisms12091769 (PMC11433644; doi:10.3390/microorganisms12091769)
Supplement: Supplementary file 1 [file microorganisms-12-01769-s001.zip › microorganisms-3162435-supplementary.pdf]

Table S1. Representative isolates and identified yeast species based on sequence of 26S rDNA D1/D2 domain.

No.	Species	Strain ID	Sample ID	Comparison of D1/D2 sequences			
				D1/D2 domain amplified product size (bp)	GenBank accession No. of type strain	Similarity (%)	Type strain
1	*Meyerozyma caribbica*	A-2	SO-YS	585	MH545919	100.00	CBS 9966
2	*Papiliotrema laurentii*	A-4	SO-YS	566	KY108739	99.82	CBS 139
3	*Clavispora* sp.	A-35	SO-YS	518	KY106454	87.31	CBS 6380
4	*Meyerozyma caribbica*	A-52	SO-YS	570	MH545919	100.00	CBS 9966
5	*Pichia manshurica*	A-72	SO-YS	576	MK394164	100.00	CBS 209
6	*Clavispora* sp.	A-76	SO-YS	534	KY106454	87.68	CBS 6380
7	*Meyerozyma caribbica*	A-117	SO-YS	605	MH545919	99.83	CBS 9966
8	*Pichia manshurica*	A-136	SO-YS	573	MK394164	100.00	CBS 209
9	*Hanseniaspora uvarum*	B-23	SO-YB	583	KY107844	100.00	CBS 314
10	*Hanseniaspora uvarum*	B-47	SO-YB	579	KY107844	100.00	CBS 314
11	*Hanseniaspora guilliermondii*	B-48	SO-YB	585	KY107797	99.66	CBS 465
12	*Hanseniaspora guilliermondii*	B-54	SO-YB	583	KY107797	99.66	CBS 465
13	*Hanseniaspora uvarum*	B-64	SO-YB	586	KY107844	100.00	CBS 314
14	*Hanseniaspora guilliermondii*	B-106	SO-YB	582	KY107797	99.66	CBS 465
15	*Hanseniaspora opuntiae*	B-137	SO-YB	565	NG055312	100.00	CBS 8733
16	*Hanseniaspora uvarum*	B-138	SO-YB	582	KY107844	100.00	CBS 314
17	*Hanseniaspora guilliermondii*	B-166	SO-YB	582	KY107797	99.66	CBS 465
18	*Hanseniaspora guilliermondii*	B-168	SO-YB	580	KY107797	99.66	CBS 465
19	*Hanseniaspora uvarum*	B-170	SO-YB	576	KY107844	99.83	CBS 314
20	*Hanseniaspora guilliermondii*	B-180	SO-YB	584	KY107797	99.66	CBS 465
21	*Hanseniaspora uvarum*	C-24	UE-CH	572	KY107844	100.00	CBS 314
22	*Lachancea thermotolerans*	C-26	UE-CH	585	XR002432225	100.00	CBS 6340
23	*[Rhodotorula] nothofagi*	C-29	UE-CH	602	NG069005	99.83	CBS 8166
24	*[Rhodotorula] nothofagi*	C-32	UE-CH	602	NG069005	100.00	CBS 8166
25	*Hanseniaspora uvarum*	C-93	UE-CH	584	KY107844	100.00	CBS 314
26	*Hanseniaspora uvarum*	C-101	UE-CH	582	KY107844	100.00	CBS 314
27	*[Rhodotorula] nothofagi*	C-123	UE-CH	600	NG069005	100.00	CBS 8166
28	*Rhodotorula graminis*	C-129	UE-CH	582	NG068963	100.00	CBS 2826
29	*Rhodotorula graminis*	C-137	UE-CH	586	NG068963	100.00	CBS 2826
30	*[Rhodotorula] nothofagi*	C-138	UE-CH	597	NG069005	100.00	CBS 8166
31	*Rhodotorula graminis*	C-144	UE-CH	585	NG068963	100.00	CBS 2826
32	*Hanseniaspora uvarum*	C-170	UE-CH	585	KY107844	100.00	CBS 314
33	*Hanseniaspora vineae*	C-247	UE-CH	582	KY107860	99.83	CBS 2171
34	*Hanseniaspora vineae*	C-268	UE-CH	581	KY107860	99.83	CBS 2171
35	*Hanseniaspora uvarum*	C-296	UE-CH	582	KY107844	100.00	CBS 314
36	*Hanseniaspora valbyensis*	D-17	UE-MR	584	KY107857	99.32	CBS 479
37	*Hanseniaspora uvarum*	D-25	UE-MR	583	KY107844	100.00	CBS 314
38	*Hanseniaspora valbyensis*	D-60	UE-MR	586	KY107857	99.32	CBS 479
39	*Hanseniaspora uvarum*	D-71	UE-MR	586	KY107844	99.66	CBS 314
40	*Hanseniaspora uvarum*	D-102	UE-MR	583	KY107844	100.00	CBS 314
41	*Hanseniaspora guilliermondii*	D-111	UE-MR	586	KY107797	99.66	CBS 465
42	*Hanseniaspora uvarum*	D-137	UE-MR	584	KY107844	100.00	CBS 314
43	*Hanseniaspora valbyensis*	D-178	UE-MR	589	KY107857	99.32	CBS 479
44	*Hanseniaspora guilliermondii*	D-192	UE-MR	591	KY107797	99.66	CBS 465
45	*Hanseniaspora valbyensis*	D-224	UE-MR	586	KY107857	99.50	CBS 479
46	*Hanseniaspora uvarum*	D-233	UE-MR	583	KY107844	99.50	CBS 314
47	*Hanseniaspora uvarum*	D-276	UE-MR	586	KY107844	100.00	CBS 314
48	*Hanseniaspora guilliermondii*	D-298	UE-MR	589	KY107797	99.66	CBS 465
49	*Hanseniaspora valbyensis*	D-323	UE-MR	591	KY107857	99.32	CBS 479
50	*Starmerella bacillaris* (synonym *Candida zemplinina*)	D-324	UE-MR	498	AY394855	100.00	PYCC 3044
51	*Hanseniaspora valbyensis*	D-356	UE-MR	587	KY107857	99.32	CBS 479
52	*Hanseniaspora vineae*	D-381	UE-MR	582	KY107860	99.83	CBS 2171
53	*Hanseniaspora uvarum*	D-387	UE-MR	588	KY107844	100.00	CBS 314
54	*Starmerella bacillaris* (synonym *Candida zemplinina*)	D-397	UE-MR	506	AY394855	100.00	PYCC 3044
55	*Hanseniaspora vineae*	D-402	UE-MR	586	KY107860	99.83	CBS 2171
56	*Starmerella bacillaris* (synonym *Candida zemplinina*)	E-3	UE-CS	501	AY394855	100.00	PYCC 3044
57	*Aureobasidium pullulans*	E-4	UE-CS	587	NG055734	100.00	CBS 584.75
58	*Hanseniaspora uvarum*	E-41	UE-CS	583	KY107844	99.66	CBS 314
59	*Hanseniaspora uvarum*	E-54	UE-CS	583	KY107844	99.66	CBS 314
60	*Hanseniaspora uvarum*	E-59	UE-CS	586	KY107844	99.66	CBS 314
61	*Hanseniaspora uvarum*	E-127	UE-CS	587	KY107844	99.66	CBS 314
62	*Hanseniaspora uvarum*	E-241	UE-CS	590	KY107844	99.66	CBS 314
63	*Hanseniaspora uvarum*	E-245	UE-CS	584	KY107844	99.66	CBS 314
64	*Hanseniaspora vineae*	E-294	UE-CS	582	KY107860	99.83	CBS 2171
65	*Hanseniaspora uvarum*	E-304	UE-CS	583	KY107844	99.66	CBS 314
66	*Hanseniaspora vineae*	E-310	UE-CS	583	KY107860	99.83	CBS 2171
67	*Saccharomyces cerevisiae*	E-324	UE-CS	584	NG042623	100.00	NRRL Y-12632

68	*Saccharomyces cerevisiae*	E-365	UE-CS	586	NG042623	100.00	NRRL Y-12632
69	*Torulaspora delbrueckii*	F-1	UR-ZG	584	XR002431970	100.00	CBS 1146
70	*Metschnikowia shanxiensis*	F-11	UR-ZG	516	NG058344	98.06	CBS 10359
71	*Torulaspora delbrueckii*	F-26	UR-ZG	590	XR002431970	100.00	CBS 1146
72	*Torulaspora delbrueckii*	F-34	UR-ZG	586	XR002431970	100.00	CBS 1146
73	*Torulaspora delbrueckii*	F-47	UR-ZG	585	XR002431970	100.00	CBS 1146
74	*Torulaspora delbrueckii*	F-48	UR-ZG	588	XR002431970	99.66	CBS 1146
75	*Metschnikowia shanxiensis*	F-84	UR-ZG	515	NG058344	98.25	CBS 10359
76	*Torulaspora delbrueckii*	F-146	UR-ZG	585	XR002431970	100.00	CBS 1146
77	*Metschnikowia shanxiensis*	F-176	UR-ZG	518	NG058344	98.07	CBS 10359
78	*[Rhodotorula] nothofagi*	G-10	UR-PB	599	NG069005	99.83	CBS 8166
79	*[Rhodotorula] nothofagi*	G-29	UR-PB	600	NG069005	99.83	CBS 8166
80	*[Rhodotorula] nothofagi*	G-45	UR-PB	603	NG069005	99.83	CBS 8166
81	*Sporidiobolus pararoseus*	G-72	UR-PB	586	NG067256	99.66	CBS 491
82	*[Rhodotorula] nothofagi*	G-144	UR-PB	589	NG069005	99.66	CBS 8166
83	*Hanseniaspora uvarum*	H-1	KN-MR	586	KY107844	100.00	CBS 314
84	*Hanseniaspora uvarum*	H-31	KN-MR	587	KY107844	99.49	CBS 314
85	*Hanseniaspora uvarum*	H-73	KN-MR	583	KY107844	100.00	CBS 314
86	*Starmerella bacillaris* (synonym *Candida zemplinina*)	H-79	KN-MR	498	AY394855	99.79	PYCC 3044
87	*Hanseniaspora vineae*	H-87	KN-MR	582	KY107860	99.83	CBS 2171
88	*Starmerella bacillaris* (synonym *Candida zemplinina*)	H-106	KN-MR	506	AY394855	100.00	PYCC 3044
89	*Hanseniaspora uvarum*	H-111	KN-MR	588	KY107844	100.00	CBS 314
90	*Hanseniaspora uvarum*	H-115	KN-MR	564	KY107844	100.00	CBS 314
91	*Hanseniaspora uvarum*	H-142	KN-MR	583	KY107844	99.66	CBS 314
92	*Hanseniaspora uvarum*	H-149	KN-MR	583	KY107844	99.66	CBS 314
93	*Hanseniaspora uvarum*	H-151	KN-MR	588	KY107844	100.00	CBS 314
94	*Hanseniaspora uvarum*	H-166	KN-MR	589	KY107844	99.66	CBS 314
95	*Hanseniaspora uvarum*	H-191	KN-MR	580	KY107844	99.66	CBS 314
96	*Starmerella bacillaris* (synonym *Candida zemplinina*)	H-200	KN-MR	503	AY394855	100.00	PYCC 3044
97	*Hanseniaspora vineae*	H-208	KN-MR	587	KY107860	99.66	CBS 2171
98	*Hanseniaspora uvarum*	H-210	KN-MR	585	KY107844	100.00	CBS 314
99	*Hanseniaspora uvarum*	H-211	KN-MR	586	KY107844	99.66	CBS 314
100	*Hanseniaspora uvarum*	H-213	KN-MR	587	KY107844	99.66	CBS 314
101	*Saturnispora diversa*	H-221	KN-MR	548	KY109545	100.00	CBS 4074
102	*Hanseniaspora uvarum*	H-238	KN-MR	588	KY107844	99.66	CBS 314
103	*Hanseniaspora vineae*	H-262	KN-MR	583	KY107860	100.00	CBS 2171
104	*Saccharomyces cerevisiae*	H-267	KN-MR	589	NG042623	100.00	NRRL Y-12632
105	*Starmerella bacillaris* (synonym *Candida zemplinina*)	H-272	KN-MR	502	AY394855	100.00	PYCC 3044
106	*Zygosaccharomyces bailii*	H-281	KN-MR	594	KY110234	100.00	CBS 680
107	*Zygosaccharomyces bailii*	H-359	KN-MR	594	KY110234	100.00	CBS 680
108	*Zygosaccharomyces bailii*	H-366	KN-MR	558	KY110234	98.92	CBS 680
109	*Zygosaccharomyces bailii*	H-367	KN-MR	593	KY110234	100.00	CBS 680
110	*Zygosaccharomyces bailii*	H-368	KN-MR	601	KY110234	100.00	CBS 680
111	*Zygosaccharomyces bailii*	H-388	KN-MR	598	KY110234	100.00	CBS 680
112	*Saccharomyces cerevisiae*	H-389	KN-MR	585	NG042623	100.00	Y-12632
113	*Starmerella apicola*	I-6	KN-CS	502	NG 075433	99.79	NRRL Y-2481
114	*Hanseniaspora uvarum*	I-14	KN-CS	583	KY107844	100.00	CBS 314
115	*Hanseniaspora uvarum*	I-15	KN-CS	583	KY107844	100.00	CBS 314
116	*Martiniozyma asiatica*	I-18	KN-CS	562	AB334112	99.64	RV60
117	*Martiniozyma asiatica*	I-23	KN-CS	562	AB334112	99.82	RV60
118	*Hanseniaspora uvarum*	I-55	KN-CS	582	KY107844	100.00	CBS 314
119	*Martiniozyma asiatica*	I-57	KN-CS	555	AB334112	99.82	RV60
120	*Martiniozyma asiatica*	I-59	KN-CS	565	AB334112	99.82	RV60
121	*Martiniozyma asiatica*	I-63	KN-CS	564	AB334112	99.82	RV60
122	*Starmerella bacillaris* (synonym *Candida zemplinina*)	I-66	KN-CS	500	AY394855	100.00	PYCC 3044
123	*Starmerella bacillaris* (synonym *Candida zemplinina*)	I-68	KN-CS	500	AY394855	100.00	PYCC 3044
124	*Starmerella bacillaris* (synonym *Candida zemplinina*)	I-128	KN-CS	500	AY394855	100.00	PYCC 3044
125	*Starmerella bacillaris* (synonym *Candida zemplinina*)	I-130	KN-CS	500	AY394855	100.00	PYCC 3044
126	*Hanseniaspora vineae*	I-133	KN-CS	589	KY107860	99.83	CBS 2171
127	*Hanseniaspora uvarum*	I-147	KN-CS	584	KY107844	100.00	CBS 314
128	*Starmerella bacillaris* (synonym *Candida zemplinina*)	I-195	KN-CS	502	AY394855	100.00	PYCC 3044
129	*Hanseniaspora vineae*	I-197	KN-CS	588	KY107860	99.83	CBS 2171
130	*Starmerella bacillaris* (synonym *Candida zemplinina*)	I-207	KN-CS	502	AY394855	99.79	PYCC 3044
131	*Hanseniaspora uvarum*	I-221	KN-CS	588	KY107844	100.00	CBS 314
132	*Hanseniaspora vineae*	I-241	KN-CS	590	KY107860	99.83	CBS 2171
133	*Hanseniaspora uvarum*	I-249	KN-CS	588	KY107844	100.00	CBS 314
134	*Starmerella bacillaris* (synonym *Candida zemplinina*)	I-266	KN-CS	500	AY394855	100.00	PYCC 3044
135	*Starmerella bacillaris* (synonym *Candida zemplinina*)	I-292	KN-CS	503	AY394855	100.00	PYCC 3044
136	*Hanseniaspora vineae*	I-321	KN-CS	582	KY107844	100.00	CBS 2171
137	*Starmerella bacillaris* (synonym *Candida zemplinina*)	I-353	KN-CS	504	AY394855	100.00	PYCC 3044
138	*Starmerella bacillaris* (synonym *Candida zemplinina*)	I-370	KN-CS	503	AY394855	100.00	PYCC 3044
139	*Starmerella bacillaris* (synonym *Candida zemplinina*)	I-378	KN-CS	515	AY394855	100.00	PYCC 3044
